# A Pragmatic Trial Evaluating the Impact of the Anumana Clinical Decision Support Tool for Guideline-Directed Management of Heart Failure (ACT-HF): Clinical trial design and methods

**DOI:** 10.1016/j.ahjo.2025.100675

**Published:** 2025-11-15

**Authors:** Francisco Lopez-Jimenez, Heather M. Alger, Sarah P. Hackett, Vinay Gundurao, Ketan Mehta, Prerak Jain, Praveen Kumar-M, Chinmay Padhye, Arjun Puranik, Kitzner Vassor, Sunil Kumar Ravi, Barbara Barry, Ranee Chatterjee, Chen Chow, Rowena Dolor, Stephen J. Greene, Grace Lin, David Rushlow, Mark Stampehl, Xuan Zhu, Samir Awasthi

**Affiliations:** a Mayo Clinic, Rochester, MN, USA; b Anumana, Inc., Cambridge, MA, USA; c nference, Inc., Cambridge, MA, USA; d Duke University School of Medicine, Durham, NC, USA; e Stormont Vail Health, Topeka, KS, USA; f Division of Cardiology, Duke University School of Medicine, Durham, NC, USA; g Duke Clinical Research Institute, Durham, NC, USA; h Mayo Clinic in Florida, Jacksonville, FL, USA; i Novartis Pharmaceuticals Corp, East Hanover, NJ, USA

**Keywords:** Heart failure, Guideline directed medical therapy, Clinical decision support, Natural language processing, Best practice alerts, Artificial intelligence, Electronic health record

## Abstract

**Background:**

Heart failure with reduced ejection fraction (HFrEF) is progressive and pervasive. Guidelines provide evidence-based recommendations to manage HFrEF, yet adherence to Guideline Directed Medical Therapy (GDMT) is low. An opportunity exists to improve adherence by delivering actionable data, reducing clinician information overload, and enhancing patient care. A Pragmatic Trial Evaluating the Impact of the Anumana Clinical Decision Support Tool for Guideline-Directed Management of Heart Failure (ACT-HF) will evaluate a clinical decision support software (CDSS) that integrates in electronic health records (EHR), automates chart review, and identifies care gaps.

**Methods:**

Anumana's Guideline Navigator is an innovative, multi-module AI-enabled CDSS with automated chart review to rapidly analyze EHR data, detect care gaps, and provide alerts for eligible patients not receiving optimal GDMT. ACT-HF, a multi-center cluster pragmatic trial, will recruit and randomize clinician participants (≤250) from 2 health systems to receive intervention software or provide usual care. The trial will evaluate outpatient care for adults with documented HFrEF and not on optimal GDMT (>2148). Outcomes will be evaluated at 90 days. Clinician participants may discuss results with patients, but patients will not have access to the CDSS.

**Results:**

Primary outcome is change in GDMT medications; exploratory endpoints include clinical outcomes, resource utilization, and usability. Subgroup analyses include health system, clinician, and patient-level characteristics associated with outcomes.

**Conclusion:**

Building on efforts to improve GDMT adherence, ACT-HF will test Anumana's Guideline Navigator in a multicenter study to evaluate outcomes and further refine the CDSS EHR integration EHR for improved clinical utility, workflow integration, and patient outcomes.

## 1 Background

Heart failure (HF) is a progressive condition and a leading cause of morbidity and mortality globally. In 2021, HF was the underlying cause in more than 85,000 deaths and contributing cause in more than 420,000 deaths; the prevalence of HF is projected to be 3 % of the US population in 2030 [1].

HF contributes significantly to healthcare resource utilization. Specifically, there were >8 million physician office visits for HF in 2019 and >1.3 million visits to the emergency department for HF in 2020 in the US [1]. The overall cost of HF is projected to increase 127 % from an estimated $30.7 billion in 2012 to $69.8 billion in 2030; with an estimated annual cost of $24,383 per HF patient; 65 % of the cost is HF hospitalizations ($15,879) [1].

The current Clinical Practice Guidelines provide evidence-based pharmacological recommendations for management of HF with reduced ejection fraction (HFrEF); Guideline Directed Medical Therapy (GDMT) [2] provides the highest level of recommendations for 4 classes of pharmacotherapy, or “quad-therapy”, for patients with HFrEF, including medications from each of the following classes: renin-angiotensin-aldosterone system inhibitors (RAASi), beta blockers, mineralocorticoid receptor antagonist (MRA), and sodium-glucose cotransporter 2 inhibitor (SGLT2i) [2]. A recent analysis of patients hospitalized with heart failure revealed that an estimated 82 % of patients with newly-diagnosed HFrEF are eligible for GDMT, but only ~15 % were prescribed quad-therapy [3].

Declines in HF mortality are primarily attributable to evidence-based approaches to managing patients with HF. GDMT is estimated to reduce cardiovascular mortality and HF hospitalization by approximately 62 % and results in 1.4–6.3 additional years of life, compared to limited conventional therapy [1].

Despite the recommendations, adherence to GDMT for HFrEF remains low [4,5]. Several potential contributing factors to this low adherence have been identified, including gaps in clinician knowledge, patience reluctance, and cost [6]. Among patients with Medicare drug plan coverage, the median monthly out-of-pocket (OOP) costs of quad-therapy is calculated at $94, compared to the $3 median monthly cost of triple-therapy with a substitution for the RAASi medication from the first-line therapy to the second-line generic [7]. Further, a recent cost analysis reported that the use of quad-therapy for HFrEF is cost effective compared to triple-therapy and double-therapy [8]. Also, a trial is currently underway to evaluate outcomes when cost is considered in a shared-decision making model [9].

A Pragmatic Trial Evaluating The Impact Of The Anumana Clinical Decision Support Tool For Guideline-Directed Management Of Heart Failure (ACT-HF) Study is designed to evaluate the clinical impact of a clinical decision support software which integrates within the electronic health record (EHR) to provide automated chart review and highlight GDMT care gaps to clinicians during routine outpatient care.

## 2 Overview of ACT-HF trial design

ACT-HF is a multi-center cluster-randomized behavioral-intervention trial that will recruit and randomize clinicians to receive access to the intervention software (Anumana's Guideline Navigator) or provide usual care.

**Fig. 1:**
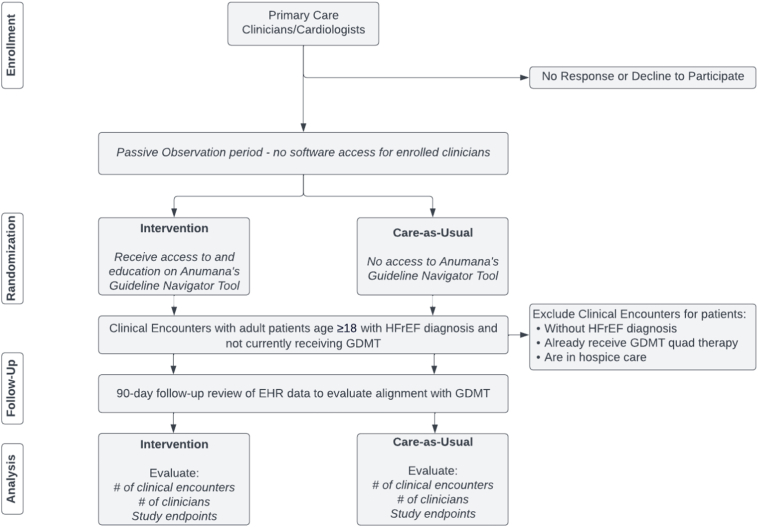
CONSORT diagram of general study design.

Clinicians (≤250) will be recruited from 2 health systems to participate in the ACT-HF Study. Participants will be clustered at the care team level within each site. The trial will evaluate the clinical care provided during >2148 eligible clinical encounters and 90 days following the index encounter. Clinicians will be consented and randomized into intervention or care as usual; patients are not recruited for this study (Fig. 1).

The primary study endpoint to be evaluated is change in GDMT medications, with exploratory endpoints evaluating other clinical outcomes. Subgroup analyses will be conducted to evaluate the health system, clinician, and patient-level characteristics associated with outcomes.

The study has received a waiver of informed consent through a central institutional review board (IRB) review (WCG Clinical Services, Princeton, NJ). The study is sponsored by Anumana, Inc. The study investigators and authors are responsible for study design, analysis, and publications.

## 3 Study population

ACT-HF is designed to evaluate the impact of an interventional software (Guideline Navigator) on clinical decision making in ambulatory care for HFrEF. Clinicians who medically manage patients with HFrEF are recruited and consented to participate in the ACT-HF study; patients are not recruited as participants in this study.

Eligible clinical encounters for the interventional study include clinical encounters with adult patients who have a diagnosis of HFrEF or a documented LVEF ≤40 %, and who are not presently on optimal GDMT. Clinical encounters will not be eligible for those patients who are in hospice care, who are pregnant, who are already receiving optimized GDMT, or for those patients who have opted out of EHR-based research.

Participating clinicians randomized to the intervention group will receive a notification when the intervention software has identified a patient record suboptimal GDMT prescribing. The clinician will choose whether and how best to inform the patient of the notification and potential changes to their care plan; individual patients will not have access to the intervention software or results. Guideline Navigator will not evaluate records of patients who do not meet eligibility criteria and no alerts will be provided.

## 4 Study intervention software

Anumana has developed an innovative AI-enabled clinical decision support system - Guideline Navigator which is a multi-module software solution with a user interface. Guideline Navigator connects with the electronic health record (EHR) through an application program interface (API) to (1) rapidly analyze patient clinical data (2) harmonize structured data, (3) curate unstructured clinical notes with natural language processing (NLP), (4) interpret patient data using a Bidirectional Encoder Representations from Transformers (BERT) model to complete an automated chart review, (5) evaluate alignment to guideline directed medical therapy (GDMT) for heart failure with reduced ejection fraction (HFrEF) using a machine learning model trained to identify eligible patients with potential gaps in care, (6) identify relevant indications and contraindications, (7) summarize potential GDMT gaps-to-care with a point-of-care user interface for clinicians to review, and (8) trigger a point-of-care alert to notify clinicians of potential opportunities to optimize clinical care for patients with HFrEF (Anumana, data on file).

**Fig. 2:**
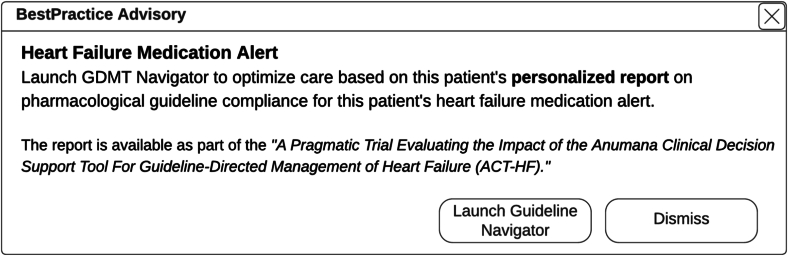
EHR-based alert display.

Guideline Navigator software is integrated within each participating health system's EHR and access is restricted to the participating clinicians. For the purposes of the ACT-HF study, Guideline Navigator will process the EHR data for eligible clinical encounters on the night prior to an appointment; clinicians may also reprocess the results at point-of-care to incorporate any updated prescription information that was made during patient check-in. A wrong recommendation will not be considered an adverse event, unless there is an event as a consequence of such. All others will be considered errors, but not adverse events. Clinicians randomized to the intervention group receive an alert (Fig. 2) for any eligible encounter with actionable results from the Guideline Navigator software; there is no alert for ineligible clinical encounters or for clinicians randomized to the standard of care group. The clinician may receive multiple alerts for a patient for a return patient who remains non-compliant with GDMT during the study period.

## 5 Study data collection

Study data will be collected from participant surveys, study software metrics, and EHR data as a read-out for clinician decision-making. Surveys were designed by experts in computer-human interaction and healthcare delivery research to quantitatively assess knowledge, readiness, usability, and acceptance of the software with questions designed to evaluate decision-making behavior, clinician sentiment towards technology-based clinical decision support solutions, and clinical inertia.

Clinician participants may discuss results with patients; however patients will not have direct contact with the study team or direct access to the ACT-HF Study software or software output. Guideline Navigator will only provide recommendations for HFrEF patients who are not currently prescribed optimal GDMT.

EHR data will automatically be collected in a privacy-preserving manner and all data will be deidentified and analyzed in the aggregate, to avoid accidental disclosure of protected health information (PHI). EHR data will be evaluated at the Index Encounter and 90-days following the Index Encounter to evaluate whether there were any changes in treatment patterns in both control and intervention groups. Pre-study surveys will be conducted to evaluate clinicians' knowledge of GDMT and their readiness to adopt the new technology. Post-study surveys will be conducted to further examine usability and acceptance of the CDS software.

## 6 ACT-HF study endpoints

Clinical outcomes data will be collected as described for the primary endpoint; participating clinicians will not be required to manually upload any clinical outcomes data, as these will be abstracted from the EHR by a clinical research coordinator. Software related data, including software-based study survey data, will be linked to clinical encounter data through a deidentified encounter ID associated with the participating provider. No identifiable patient data will be uploaded into the electronic data capture (EDC) system.

Outcomes of interest in the ACT-HF study are shown in Table 1. The primary outcome of the ACT-HF Study is to evaluate the total number of GDMT-HF-recommended HFrEF medications prescribed at 90-days post clinical encounter, per patient, compared to care-as-usual (control arm). Exploratory endpoints will be evaluated in three categories: clinical outcomes, intervention software CDS outcomes, and patient outcomes. At the completion of the study, subgroup analyses will be conducted to evaluate the impact of the Intervention software by factors such as clinical setting, clinician factors, and patient factors.

**Table 1:** ACT-HF study endpoints.

ACT-HF Study - Primary endpoints
• Number of GDMT-recommended medications for HFrEF prescribed at 90-days post clinical encounter, per patient, compared to care-as-usual (control arm)


ACT-HF Study - Exploratory endpoints		
Clinical outcomes	Software usability outcomes	Patient outcomes
• Change in patient NYHA Class from baseline through observational period • % of patients taking optimal GDMT-HF therapy based on software-identified indications and lack of contraindications • Time to start of new GDMT-HF medication class • Dosage of GDMT-HF-recommended quad therapy HFrEF medications reached • % of patients for whom at least one GDMT-HT medication was prescribed.	• User engagement with the software, including session counts, session duration, and report button click counts. • Percent of notification prompts that are accepted or dismissed, at the clinician level. • Clinician experience with software (survey-based) o Cognitive behavioral, and contextual factors related to clinician satisfaction, acceptance, and use of the software o Reasons for rejecting, modifying, or ignoring recommendation o Barriers and facilitators to use	• Changes in BNP or NT-pro-BNP, blood potassium, blood creatinine, and estimated glomerular filtration rate • Hospitalizations o Heart Failure o All-cause • Mortality o Heart Failure o All-cause • Indicators of resource utilization o Ordering of echocardiogram, time from index encounter, and echocardiogram results o Outpatient appointments o Specialist visits o Hospital days o Emergency room visits o 30 day readmissions following a heart failure hospitalization


ACT-HF Study - Safety endpoints
• Incidences and event rates of study-related adverse events (AE), compared to control arm • Incidences and event rates of study-related Severe Adverse Events (SAE), compared to control arm

Adverse events (AE) and severe adverse events (SAE) will also be reported throughout the duration of the study, as applicable. For the purposes of the ACT-HF study, adverse events include death, side effects or allergies to a medication that was started due to a failure of the software to identify prior side effects or allergies and the clinician fails to check for prior side effects or allergies, or other reportable events. ACT-HF is a minimal risk study and AE/SAEs are not anticipated. If any AE/SAE occurs during the study, clinician participants are asked to notify the Site PI or designee to report the event within 24 h of knowledge of the event.

## 7 Discussion

The 2022 Clinical Practice Guidelines for Heart Failure Management provide the strongest class of recommendation for optimizing GDMT quad-therapy for patients with HFrEF, based on endpoint data from several randomized clinical trials [2]. The 2022 HF Guideline added the SGLT2i class of medications to the previous GDMT which recommended three classes of medications (triple-therapy) based on evidence available at the time: RAASi, Beta Blockers, and MRA [10]. In a real-world study of GDMT triple-therapy adherence, incomplete adherence to GDMT triple-therapy was associated with 29 % excess mortality risk in two year follow up [11].

In addition to the classes of medication in GDMT, timely achievement of the target dose of the classes of medications is an important factor in outcomes for patients with HFrEF. A multinational study of Utilization of Dapagliflozin and Other Guideline Directed Medical Therapy in Heart Failure Patients: (EVOLUTION-HF) reported that the mean time from HF hospitalization to GDMT initiation were longer for novel GDMT classes compared to other GDMT classes in Japan, Sweden, and the United States [12]. Further, an analysis of nearly 5000 patients without contraindications in the Change the Management of Patients with Heart Failure (CHAMP-HF) registry demonstrated reduced mortality with target dosing of RAASi and Beta Blocker Medications [13]. These data highlight the importance of managing the classes, timing, and quantities of GDMT medications.

Despite substantial evidence supporting GDMT for HFrEF, medical management of HFrEF patients falls short and adherence remains low. An analysis of data from the PINNACLE (Practice Innovation and Clinical Excellence) Registry of ambulatory care in heart failure reported that 60.7–71.4 % of ambulatory patients with HFrEF received Beta Blockers and RAASI medications from 2013 to 2017 [14]. In the CHAMP-HF Registry, only 1 % of patients were receiving the standard of care GDMT triple-therapy at the time of the study [15]. Among HF patient groups, disparities in care also exist among patients of color, despite high HF prevalence [16,17] and among females compared to males [18].

Several recent studies have evaluated novel methods and tools to improve medical management and medication adherence in HFrEF through patient/provider interventions, including: PRagmatic trial Of Messaging to Providers about Treatment of Heart Failure (PROMPT-HF) [19], Building Electronic Tools to Enhance and Reinforce Cardiovascular Recommendations for Heart Failure (BETTER CARE-HF) [20], Safety, tolerability and efficacy of up-titration of guideline-directed medical therapies for acute heart failure (STRONG-HF) [21], and Safety And Efficacy Of Virtual Care Team Guided Therapeutic Optimization During Hospitalization In Patients With HFrEF (IMPLEMENT-HF) [22]. Specifically, PROMPT-HF reported a > 40 % greater GDMT prescribing among clinicians who received a point-of-care EHR-based alert, compared to usual care in a single ambulatory care center [19]. Similarly, BETTER CARE-HF compared the effectiveness of an automated alert or message to usual care in MRA prescribing patterns in an outpatient cardiology practice; and reported that the automated alert was more effective than both message and usual care [20]. Investigators in the STRONG-HF study reported that rapid uptitration of GDMT and closely following hospitalized HF patients post-discharge was effective at reducing 180-day all-cause mortality and heart failure readmission rates compared to usual care across health systems in 14 countries [21]. Lastly, IMPLEMENT-HF demonstrated that a virtual care-team approach which reviewed charts of patients hospitalized with HFrEF and provided recommendations for GDMT optimization increased the use of β-blockers, ARNI, MRA, and triple-therapy compared to usual care [22].

The ACT-HF Study and the Guideline Navigator software were designed with the learnings from these studies as guides to drive improvement in GDMT adherence with novel methods, by leveraging a novel end-to-end software platform, Anumana's Guideline Navigator, and a point-of-care alert to notify clinician participants of the potential deviation from GDMT. ACT-HF is a multi-center study to evaluate the clinical effectiveness of Guideline Navigator, which automates chart review of both structured and unstructured data in the EHR to synthesize and present the key data in medication management: diagnoses, current medications, indications, and contraindications. ACT-HF will also evaluate study endpoints and subgroup analyses to further refine the Guideline Navigator software for improved clinical utility, workflow integration, and patient outcomes. We project that the observational period will complete in May 2025 with 90-day data readout and primary analyses available in late 2025. Analysis of endpoints and subgroup analyses may be presented later in 2026. Subgroup analyses may include evaluation of outcomes by site-level, clinician-level, or patient-level characteristics, examples may include site geography, participant professional characteristics, or patient medical history.

There are limitations to the ACT-HF study that must be considered. First, the study success depends on the successful integration of the study software within each study site's secured EHR system. Because of the sensitive nature of patient EHR data, this process requires several reviews and approvals before the software environment can be made available for testing. Also, study outcomes depend on user engagement with the software. Intervention group clinicians will be trained on the Guideline Navigator software at consenting but are not required to use the study software for clinical decision making. To address this potential bias, the study will include a subgroup analysis comparing study outcomes among Low Adopters and High Adopters of the software. Additionally, the ACT-HF study is designed to evaluate the feasibility of deploying an EHR-integrated software, Guideline Navigator, to identify and remediate non-compliance to GDMT; the primary endpoints do not explore cost considerations, implications, and barriers of additional prescribing. Before this or similar tools are deployed in broader clinical setting beyond this randomized study, a thorough evaluation of stakeholder costs is warranted to ensure that the software does not exacerbate health disparities.

In this first-of-its-kind, multicenter analysis, the ACT-HF Study will evaluate the impact of a novel CDS software to impact optimal GDMT for HFrEF, including clinician experience and engagement with the software. ACT-HF will also evaluate clinician acceptance of the Clinical Practice Guidelines, as well as self-reported acceptance of the CDS. The goal of the intervention is to (1) increase GDMT adherence, (2) reduce health disparities by helping to identify opportunities at point-of-care to identify patients or patient groups who may be at risk of suboptimal GDMT through an automatic system that will not be influenced by socioeconomic status, gender, race or ethnicity, and (3) reduce cognitive burden in busy clinical settings across geographically diverse clinical settings.

## Funding support

The study is sponsored by Anumana, Inc. The study investigators and authors are responsible for study design, analysis, and publications.

## CRediT authorship contribution statement

**Francisco Lopez-Jimenez:** Writing – review & editing, Supervision, Methodology, Investigation, Conceptualization. **Heather M. Alger:** Writing – review & editing, Writing – original draft, Project administration, Methodology, Investigation, Funding acquisition, Conceptualization. **Sarah P. Hackett:** Writing – review & editing, Project administration, Methodology, Investigation, Funding acquisition, Conceptualization. **Vinay Gundurao:** Writing – review & editing, Supervision, Software, Project administration. **Ketan Mehta:** Writing – review & editing, Software, Methodology, Conceptualization. **Prerak Jain:** Writing – review & editing, Validation, Software. **Praveen Kumar-M:** Writing – review & editing, Validation, Software. **Chinmay Padhye:** Writing – review & editing, Validation, Software. **Arjun Puranik:** Writing – review & editing, Methodology, Formal analysis, Data curation. **Kitzner Vassor:** Writing – review & editing, Software. **Sunil Kumar Ravi:** Writing – review & editing, Validation, Software. **Barbara Barry:** Writing – review & editing, Methodology, Investigation. **Ranee Chatterjee:** Writing – review & editing, Supervision, Investigation. **Chen Chow:** Writing – review & editing, Supervision, Investigation. **Rowena Dolor:** Writing – review & editing, Supervision, Investigation. **Stephen J. Greene:** Writing – review & editing, Supervision, Investigation. **Grace Lin:** Writing – review & editing, Supervision, Methodology, Investigation. **David Rushlow:** Writing – review & editing, Methodology, Investigation. **Mark Stampehl:** Writing – review & editing, Methodology, Investigation, Conceptualization. **Xuan Zhu:** Writing – review & editing, Methodology, Investigation. **Samir Awasthi:** Writing – review & editing, Supervision, Project administration, Methodology, Investigation, Funding acquisition, Conceptualization.

## Human ethics and consent to participate declaration

As an implementation science study designed to evaluate the impact of a novel clinical decision support software on clinician decision making, ACT-HF will recruit and consent clinicians to participate in the study; ACT-HF will not recruit individual patients to participate in the study and patients will not have access to the CDS study software. There will be no direct contact with individual patients during this study and the study has received a waiver of informed consent through a central institutional review board (IRB) review (WCG Clinical Services, Princeton, NJ).

## Declaration of competing interest

Authors Alger, Hackett, Gundurao, Mehta, Jain, Kumar-M, Padhye, Puranik, Vassor, Ravi, and Awasthi are, or were, affiliated with Anumana, Inc. a Cambridge, MA based company that develops software as a medical device artificial intelligence-enabled algorithms to detect cardiovascular conditions. All other authors have reported that they have no relationships relevant to the contents of this paper to disclose.

Dr. Greene has received research support from the Duke University Department of Medicine Chair's Research Award, American Heart Association, Amgen, AstraZeneca, Bayer, Boehringer Ingelheim, Bristol Myers Squibb, Cytokinetics, Merck, Novartis, Otsuka, Pfizer, and Sanofi; has served on advisory boards or as consultant for Amgen, AstraZeneca, Bayer, Boehringer Ingelheim, Bristol Myers Squibb, Corcept Therapeutics, Corteria Pharmaceuticals, CSL Vifor, Cytokinetics, Eli Lilly, Lexicon, Merck, Novo Nordisk, Otsuka, Roche Diagnostics, Sanofi, scPharmaceuticals, Sumitomo, and Tricog Health; and has received speaker fees from AstraZeneca, Bayer, Boehringer Ingelheim, Cytokinetics, Lexicon, Novo Nordisk, and Roche Diagnostics.

Dr. Lopez-Jimenez is a member of the scientific advisory board for Anumana, and a co-inventor of Anumana's ECG-AI LEF algorithm.
